# Two Sides of the Coin: Unveiling the Socioeconomic Impacts of Land Expropriation on Kigali Residents

**DOI:** 10.1007/s12132-024-09516-3

**Published:** 2024-05-22

**Authors:** Gideon Baffoe, Vincent Manirakiza, Ernest Uwayezu

**Affiliations:** 1 Department of Environment and Geography, University of York, 290 Wentworth Way, Heslington, York YO10 5NG UK; 2 College of Education, University of Rwanda, P.O. Box 55, Rwamagana, Kigali Rwanda; 3 Centre for Geographic Information Systems and Remote Sensing (CGIS), University of Rwanda, Nyarugenge Campus, KN 73 St., P.O. Box 3900, Kigali, Rwanda

**Keywords:** Expropriation, Assets, Livelihood, Land, Kigali

## Abstract

In a bid to become global cities and centers of innovation, many African cities have embraced rapid physical transformation as the default urban development paradigm. However, this development mantra is exacting a significant social cost. At the core of this physical transformation lies land expropriation, granting governments the power to accumulate land in the name of public interest. Focusing on Kigali as a case study and employing the livelihood asset framework and snowball sampling technique, we examine the impact of land expropriation on asset endowment and the well-being of affected individuals. Results reveal a complex picture. Expropriation dispossesses individuals of their primary productive livelihood assets – physical, financial, and social – leading to impoverishment. This practice not only risks exacerbating the poverty cycle but also undermines Kigali's reputation as a "Model City of Africa." Rebuilding these assets has proved challenging for most, with insufficient compensation often used for subsistence rather than productive activities. To cope with the new life, livelihood diversification emerges as the primary resilience-building strategy. However, the study reveals that expropriation does not always result in the deprivation of productive assets. In cases where individuals receive adequate compensation, expropriation appears to facilitate social mobility through improved housing and investments in profitable ventures. Nonetheless, expropriation, the study concludes, yields significant and varied socio-economic impacts, and addressing these would require integrated and multifaceted measures. We advocate for a revised compensation package by the government to mitigate asset deprivation. Additionally, we recommend government investment in affordable housing, alternative livelihood options, promotion of participatory planning, facilitation of capital acquisition for small-scale businesses, and coaching for affected property owners on investment strategies and livelihood reconstitution post-expropriation.

## Introduction

Rapid urbanization does not only drive city structure but also dictates the form that urban policies will take. Urbanization continues to provide traction for most urban woes, including informal settlement emergence, land speculation, poverty, and pollution (World Cities Report, 2020; Baffoe & Roy, 2022; Baffoe, 2023). These intricate challenges put pressure on urban land, making it a scarce commodity and affecting not only urbanites but also national governments’ efforts to expand and transform urban infrastructure in various shapes (World Bank, 2020; de Bruin et al., 2021). The criticality of access to land has been widely recognized under many binding and nonbinding international human rights protocols as an implied human right (De Schutter, 2010). Land is required for the optimum enjoyment of human rights, particularly in access to livelihood, housing, and the right to self-determination (Tura, 2018).

In a bid to manage urbanization and be regarded as modern cities, many cities in the developing world have embraced land expropriation as a major land-use strategy to acquire lands for developmental projects (e.g., roads, schools, and hospitals) (Ong, 2020; Tura, 2018). Expropriation, also known as eminent domain, is the compulsory acquisition of land by a government for the public interest (World Bank, 2015a, b). Although this activity is restricted to cases where land is needed for public-based developments, the observation is that it has been abused as many governments hide under the guise of the law to acquire lands for private sector-led development (World Bank, 2015a, b). In China, for instance, a recent survey indicates that between 2012 and 2016, 1800 urban villages in 17 provinces were expropriated by the government for infrastructural development. Out of this accumulation, 12%, 10%, and 7% of the land were earmarked for factories, industrial parks, and commercial districts, respectively (Landesa Rural Development Institute, 2018). It is estimated that in 2010, 65% of mass incidents in China, including riots and civil unrest, were attributed to land expropriation (Landesa Rural Development Institute, 2012). Statistics indicate that 50 million people have lost their lands because of expropriation and out of this figure, more than 30 million are living in poverty (Qin et al., 2012 cited in Wang et al., 2019). Underlying eminent domain activities compulsory acquisition is the desire to boost local economic growth, and job creation (World Bank, 2015a, b; Zhao et al., 2022). While some scholars argue that nature reserves-based expropriation has the potential to promote environmental conservation and the development of biological species, (Chen et al., 2019; Debonne et al., 2019), the seeming consensus is that land expropriation results in the dispossession of properties and displacement, which go a long way to engineer food insecurity, but also the destruction of social capital (Wineman & Jayne, 2017). The act also impinges on the health outcomes of victims (Marco-Thyse, 2006), in addition to engendering conflicts between governments and the general populace (Ma et al., 2016; Li & Hu, 2018).

Research in the subfield of expropriation has focused on major areas, including rights (Bao et al., 2016; Li et al., 2019), economic returns (Ding, 2007), social organization (Pu & Chen, 2009), conservation (Castro-Arce & Vanclay, 2020), health (Wang et al., 2019; Zhao et al., 2022), trust and conflict (Zhao and Xie, 2022). This study aims to build on existing research by critically examining the socio-economic impacts of land expropriation using Kigali as a case study, with a specific focus on asset taking, compensation, coping strategies and the subsequent well-being of affected households. It seeks to investigate whether expropriation enhances living conditions or puts affected individuals in any situation of deprivation.

Kigali is one of the cities in Africa experiencing rapid urbanization and transformation (Goodfellow, 2022). The city exemplifies the challenges and opportunities arising from expropriation-induced urban transformation. This phenomenon positions land as a precious commodity, particularly in peri-urban areas where it is a major livelihood asset. Given its scarcity, the land was also a major source of conflict in the past (Republic of Rwanda, 2015). Access, therefore, remains a major developmental challenge that governments and citizens grapple with. Regularization attempts include the nationwide Land Tenure Reform( LTR) program, which sought to demarcate, adjudicate, and register individual land holdings between 2009 and 2013. The program is believed to have enhanced land security and increased livelihood opportunities in rural areas (Focus on Land in Africa, 2019). To achieve the modernization agenda, the government has adopted a comprehensive City Master Plan (Republic of Rwanda, 2015) to streamline spatial planning while regulating urbanization. Key to the master plan is the expropriation law, which empowers the government to acquire private lands for public use. The expropriation effects since its inception in 2013 have been far-reaching, but existing analysis has done little to understand how the exercise has impacted the well-being of affected individuals. Existing studies have focused largely on the legalities of expropriation (e.g., Rose et al., 2016; Rwanda Civil Society Platform, 2017; Uwayezu & de Vries, 2019, 2020). For instance, while Rose et al., (2016) looked at the law and its outcomes and the way forward, Uwayezu and de Vries (2019) considered the spatial justice aspect of the law. To date, little evidence exists to understand, for instance, the challenges and coping strategies adopted by asset-deprived households of expropriation. This study seeks to contribute empirical evidence to fill the existing gap. The results and recommendations from this study would be relevant in shaping future policies about post-expropriation well-being and asset accumulation initiatives in Rwanda and beyond.

The study is divided into five sections. The next section contextualizes land expropriation in Kigali. Section three provides the methods, including a short profile of Kigali as a ‘model city’. Section four presents the results while section five concludes the study.

## Assets Theoretical Framework

Assets are critical resources as they form the basis for successful livelihood attainment and improved well-being. The asset framework presents poverty as a multi-dimensional problem, which is an extension of the traditional income approach (Lu & Xu, 2016). Assets may be tangible (e.g., land, tools, and stores like savings) or intangible, which are inherent qualities such as rights, social capital, capacity, and values (Nel, 2015). Assets are grouped into five categories: natural, social, human, physical and financial assets (Morse & McNamara, 2013; UNDP, 2017). Assets influence households’ decisions on which livelihood strategies and objectives to pursue within a particular context (Ding et al., 2018). Financial assets include resources such as savings and remittances, credit facilities, and income from labour (OECD, 2013; Bernahu & Woldemikael, 2022). Although they are the most limiting assets for the urban poor, access to such resources is critical for the development and accumulation of other assets (Baffoe & Matsuda, 2018; Wang et al., 2021). Human capital comprises access to training and education, skill, knowledge, good health, and the ability to labor (Baffoe & Matsuda, 2017). A lack thereof amounts to the underutilization of other assets (Boateng, 2013; Brunting et al., 2013). Physical assets are those resources critical for engaging in productive activities. These include land, tools, houses, shops, and communal resources such as roads, sanitation facilities, markets, and electricity (Brunting et al., 2013; Bernahu & Woldemikael, 2022). Existing evidence shows that access to physical assets, particularly in the urban context, allows the poor to be economically viable, thereby escaping poverty (Fontana, 2016). Social assets are resources that are embedded in relationships, including ties, networks, group membership and affiliations. These are intangible resources that people draw on for diverse livelihood pursuits. Natural assets, on the other hand, are resource stocks, including water, plants and trees, and land (Daily et al., 2011; OECD, 2013).

Assets are critical for poverty reduction, particularly in the urban informal context. Given that most residents in informal settlements are unsalaried workers, the ability to improve their well-being is contingent upon their access and accumulation of diverse assets and the return they generate (Moser & Dani, 2008). To this end, urban interventions aimed at building the capacity of the poor to cope with the adverse impacts of shocks mostly target improving livelihoods and asset accumulation (Nel, 2015). Although evidence shows that there is a linkage between livelihood asset possession and well-being attainment among the urban poor (Soma et al., 2020), there is a limited understanding, especially in the African context, of how expropriation affects the livelihood asset base of the poor. Using this asset theoretical perspective, this study aims to contribute to filling this literature gap.

## Contextualization: Land Expropriation and its Legal Framework in Kigali

Expropriation in Rwanda dates to the pre-independence time when it was governed by the Decrees of 5/02/1932 and of 30/07/1953 (Decrees of 1932 and 1953). The decrees were later modified by the Decree of 24/07/1956 (Reyntjens, 1980). Expropriation has been largely employed by the Government of Rwanda as a land management tool to control rapid urbanization and population growth against a backdrop of poor urban planning and regulations (Goodfellow, 2014). Post-independence constitution of 2003 gave more power to the government to initiate expropriation on the grounds of public interest. It is believed that the embrace was largely influenced by international instruments such as Article 14 of the African Charter of Human and Peoples Rights and Article 17 of the Universal Declaration of Human Rights, which stipulates that no person shall be arbitrarily deprived of his property. Article 29 of the 2003 Constitution of the Republic of Rwanda (revised in 2015) also upholds the right of individuals to own property. The international laws and standards particularly emphasize that “just compensation” must be paid to all affected parties, and that the state must put in place a transparent procedure to guide all processes, including property valuation, dispute resolution, and compensation (Rose et al., 2016). Just compensation value is the value of the expropriated property, which is determined at market price (Tagliarino et al., 2018). The effort to support the constitutional principle in post-independent Rwanda saw the promulgation of Decree-Law no 21/79 of July 23rd, 1979, which sought to control the act of expropriation (USAID, 2014).

The latest law governing expropriation in Rwanda is the 2021 Land Law and 2007 Expropriation Laws. The 2007 Law was amended in 2015 with Law No. 32/2015 of 11/06/2015 (Government of Rwanda, 2015). Articles 2 and 3 of Law No 18/2007 of 19/04/2007 speak to expropriation defining it as the “taking of the private property in the public interest aiming at development, social welfare, security and territory integrity” (Law No. 18/2007 of 19/04/2007). The Expropriation Law clarifies the rights of individuals in the process of expropriation. Thus, it seeks to make clear the valuation and compensation processes. According to the law, individuals affected by any expropriation exercise are entitled to “just compensation” for losing their property (Government of Rwanda, 2015). To enforce this, the law encourages that funding for compensation and other related costs needs to be available before any step is taken by expropriating agencies (Rose et al., 2016). Practical procedures that provide an impetus for “just compensation” include the use of updated reference prices, determined at market value, and the triggering of counter-assessment of the proposed compensation value in a situation where affected people are not satisfied (Government of Rwanda, 2015). The decision on the option or form of compensation is a prerogative of the expropriating agencies, irrespective of whether expropriation is initiated for public or private interest. However, there is always room for negotiation, particularly when a private investor seeks to actualize the master plan. Here, expropriation can be initiated as an act of public interest when negotiations between property owners and investors fail (Government of Rwanda, 2015). In relation to the Land Law, while Article 5 speaks to the right to own private land (either acquired by official title or customarily), Article 34 of the law protects private ownership rights, stipulating that “the State recognizes the right to freely own land and shall protect the landowner from being dispossessed of the land whether totally or partially, except in the case of expropriation due to public interest”. Other supporting provisions of expropriation include Law No 43/2013 of 16/06/2013, which governs land in Rwanda, Article 2 (140) Law No 17/2010 of 12/05/2010, which establishes and controls the real property valuation profession in Rwanda, and Ministerial Order No 001/16.00 of 23/11/2009, which determines the reference land prices in the city of Kigali (Ministerial Order No. 001/16.00 of 23/11/2009).

To uphold good practice, and in line with international standards, the Expropriation Law of 2007 clearly defines projects that can warrant the exercise under public interest, including but not limited to roads and railways, water dams and electric lines (Rose et al., 2016). Under the Law, ministries are empowered to undertake expropriation for large-scale projects. Here, the Minister of Natural Resources or the Prime Minister has the authority to approve expropriation activities. At the district or the city level, Executive Committees are permitted to put forward expropriation proposals within their hegemony. More importantly, Land Committees at the District and national levels have the responsibility to assess every expropriation application to make sure that all legal requirements are fulfilled (Rose et al., 2016). One thing that the law does not grant Kigali city and its associated districts is the power to undertake expropriation for a real estate agency whose aim is to develop commercial properties. However, in practice, the clause has been barely observed because the opposite has become more pronounced, with the cases of Rugarama and Kangondo being examples (Hudani, 2020). Real estate agencies continue to lobby government agencies to initiate expropriation on their behalf in prime areas for property development. The sad thing is that most of these estate properties are expropriate for the interest of few urbanites at the expense of the majority poor and low-income groups (Baffoe et al., 2020). The observation in Rwanda is that the power of eminent domain has mixed applications; both in public and private interests, but without prior consultation with property owners in most cases of private interests (Goodfellow, 2014).

Expropriation has been responsible for the current urban transformation in Kigali, through the implementation of the Master Plan crafted in 2013 and revised in 2019, with the underlying aim to reshape the city (Hudani, 2020; Goodfellow, 2022). The cases of expropriation have been on the ascendency after the 2007 Law, with an estimated 60.5% occurring after 2012 (Rose et al., 2016). Specific projects include roads, affecting 55% of all expropriated households, followed by dams (14.6% of all expropriated households), commercial facilities (10.5%), water and electricity infrastructure (7.2%), and public service buildings (6.8% of expropriations) country-wide (Rose et al., 2016). Its impacts, however, have been far-reaching, with issues of compensation which does not allow for acquiring other properties and dissatisfaction being rife in the capital city. For instance, in the case of Kangondo II, property owners bitterly complained against the decision by Kigali city authorities and Gasabo district to resettle them in shared residential apartments, without prior consultation and consent (Hudani, 2020). A recent study by Rwanda Civil Society reports gross dissatisfaction among property owners because in most cases they are given limited time to negotiate compensation, while some are not consulted at all before expropriation (Rwanda Civil Society Platform, 2017). However, little has been done on how the exercise has impacted the assets of its victims. Further research is, therefore, needed in this area.

## Methodology

### Kigali ‘the Model City’ as a Case Study

**Fig. 1:**
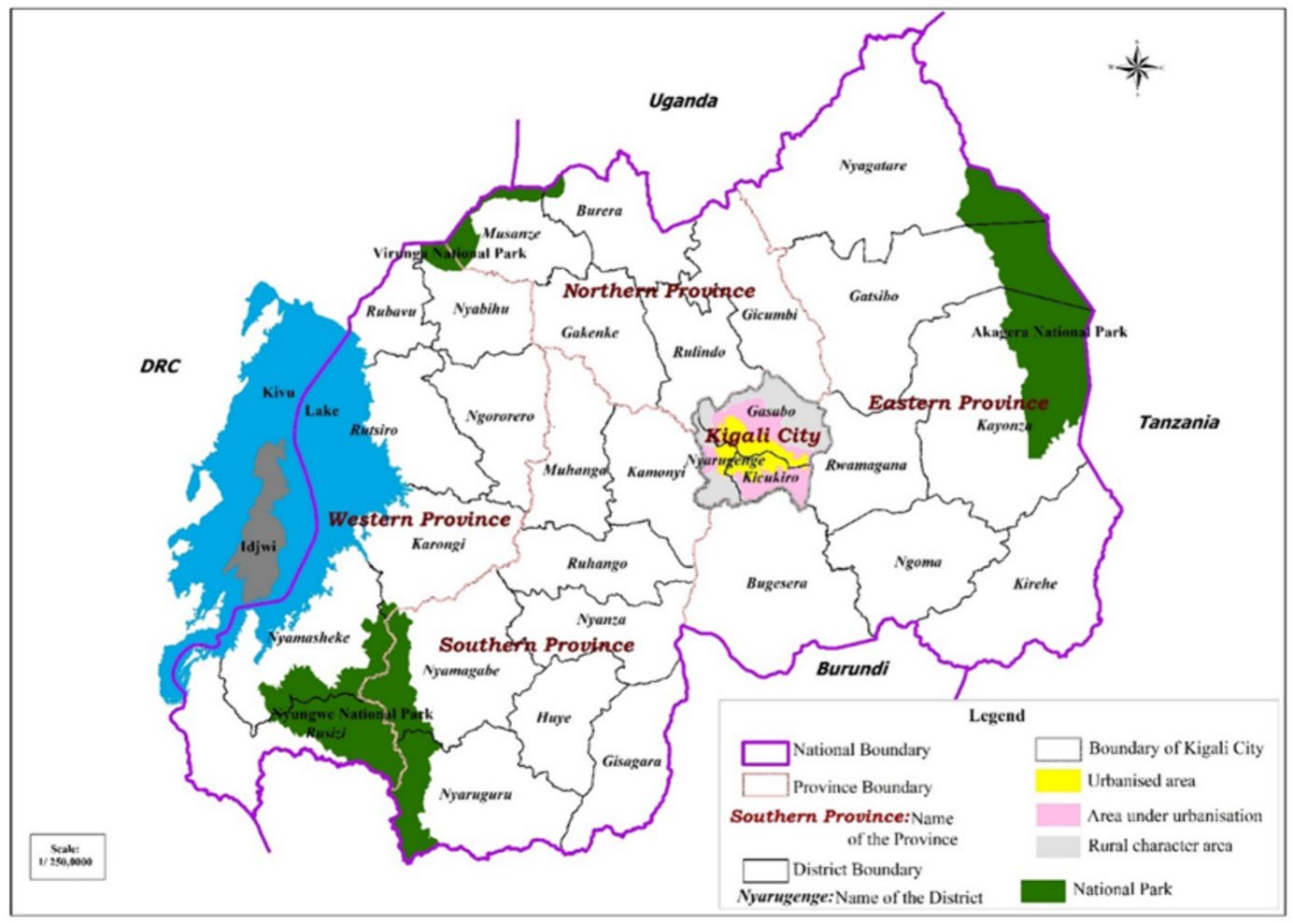
The location of Kigali city in the national context Source: Uwayezu & de Vries, 2019

Kigali City is composed of three major administrative districts: Gasabo, Kicukiro, and Nyarugenge, which are among 30 districts that make Rwanda. Figure 1 shows the boundary of Kigali city with its constituent districts, comprising three main spatial patterns in relation to land development: the urbanised zone, the zone under urbanisation and the urban fringe.

The city has population of 1,745,555, with a density of 2,401 inhabitants per square kilometer. The youth population (between 16–30 years) represents 33% of the total population, with women forming the majority with slightly over 50% (National Institute of Statistics of Rwanda, 2023). Kigali has experienced an unprecedented transformation over the past two decades, earning the nickname the ‘Singapore of Africa’ (Manirakiza et al., 2019). Given its transformational achievement, the city won the UN-Habitat Scroll of Honor Award in 2008 for its leading role in building a model, modern city, characterized by zero tolerance for plastics, improved sanitation, and crime prevention. Kigali’s position as a ‘model city’ is also exemplified by being the cleanest city in Africa; its role in championing investment and economic growth; the establishment of ambitious development plans, and the monthly community work to clean the city (Umuganda) (Bafana, 2016; Goodfellow, 2022; Goodfellow & Smith, 2013). The city's leadership has been proactive in implementing strategic plans to accommodate the growing population while maintaining environmental sustainability and attracting local and foreign private investors (Goodfellow, 2022). Through this process, the city has embraced technological advancements to enhance the quality of life for its residents. Initiatives such as smart infrastructure, digital governance, and connectivity contribute to Kigali's image as a forward-looking and technologically advanced urban center in Africa.

The rapid growth of Kigali, however, has been accompanied by rising unplanned settlements, which represent 80% to 90% of the current housing stock. Unplanned settlements are largely occupied by rural migrants. The current estimate has it that about 43,436 affordable housing units will be needed in the next seven years to bridge the housing deficit (Esiara, 2015). Given the increasing number of informal settlements and an incessant demand for affordable housing, urban planning has become a major developmental challenge that city authorities continue to grapple with (USAID, 2014). Attempt to regularize spatial planning has seen the government instituting land use master plans, which started with the Kigali City Conceptual Master Plan – promulgated in 2007 but approved in 2013. Also worthy of note are the National Urban Housing Policy (2008) and Human Settlement Policy (2009), which aimed to enhance appropriate zoning and informal settlement upgrading and city modernization, respectively (USAID, 2014). With a commitment to good governance, the government has outlined pragmatic modalities aimed at streamlining land management in urban Rwanda. In particular, the government has reviewed Organic Law N° 08/2005 of 14/07/2005, which determines the use and management of land. This exercise was to make the law an ordinary law, thereby complying with constitutional provisions (Republic of Rwanda, 2015). The new land Law (N° 43/2013 of 16/06/2013) is largely regarded as the perfect instrument to consolidate all dimensions of existing land policies, particularly that of 2004.

In a bid to operationalize the various legislative instruments, urban expropriation has become pervasive. The compulsory acquisition of private property by the government for the advancement of public goods has cast a dent in the government’s modernization agenda. Expropriation has been accompanied with the demolition of structures in the core urban zones which are informally developed to pave way for modern designs and development, including green spaces, as suggested in the master plans (Hudani, 2020). Scholars and some commentators seem to concur that the overall exercise is rigid and largely focused on city aesthetics, with little attention given to the actual needs of the people. The ramifications have been far-reaching, particularly when the few elites and investors keep lobbying expropriating agencies to consider their [private] projects as public interest (Goodfellow, 2014, 2022).

### Study Approach: Qualitative Case Study

To study how people perceive the impact of expropriation on their assets, a qualitative case study approach was deemed the most suitable approach. Given that the subject under investigation has subjective elements, a case study offers the best approach to unearth the nuances, which is important in appreciating the significance that people attach to a social issue (Baffoe et al., 2020; Creswell, 2009). Gummesson (1988) argues that the approach allows a comprehensive understanding of a phenomenon, dwelling on one’s ability to observe and evaluate different shades of social issues and how they are interlinked together. Yin (2009) adds that these interlinkages are critical in placing contextual issues in perspective. The approach is deemed an appropriate strategy as it offers the researchers the opportunity to understand the contextual socioeconomic, cultural, and political factors that shape the experiences of expropriated victims in Kigali.

### Data Collection and Analysis

The study collected data from 21 participants across the three districts (Gasabo, *n* = 9, Nyarugenge, *n* = 7, and Kicukiro, *n* = 5) of Kigali through in-depth interviews. The selection was based on the number of years living in Kigali (five years and above) and at least one-time experience of expropriation process. In terms of gender, 12 males and 9 females aged 18 and above were interviewed about their experiences with expropriation. The study adopted the snowball technique in sampling the participants. Dwelling on the researchers’ networks in each district, a household that has experienced expropriation was identified in the new residential sites for interview. These households were then asked to suggest other expropriated households whom they know for interview. The approach proved to be very effective in identifying the target population. The interview was conducted in English and the local language (Kinyarwanda) and focused on major issues, including the livelihood options before and after the expropriation, the livelihood opportunities and challenges driven by the expropriation process, and the strategies adopted to cope with the expropriation challenges.

To support the interview data, the researchers also conducted field observations in the resettled communities to understand the general conditions. Here, pictures that are reflective of the impact of the expropriation process were taken to provide contextual information. Field notes were also taken to provide additional information. Other sources of data include journal articles and books, working papers, and reports on the expropriation in Kigali City. In analysing the data, we followed the thematic-based approach. The data was first transcribed and coded in Microsoft Excel. The researchers then individually identified themes and patterns related to the major issues identified above. We then compared notes to ascertain similarities and differences. The notes were very similar (more than 90%) and showed that we all had a similar understanding of the emerging themes. No major disagreement was recorded at this stage. The last process was producing the narrative following the study objectives. These processes were followed to ensure that the results were transparent and robust. Ethical clearance for the study was sought from the Universities of York and Rwanda and the Kigali City office.

## Results

### Asset Loss and Dispossession

Employing the asset framework, the study sought to investigate the most dispossessed assets among expropriated victims. The data (Table 1) revealed that physical, financial, and social assets are the most dispossessed assets with a total and sharp decline. From the data, this is represented by 100%, 85%, and 70% response rates, respectively. The loss was largely attributed to the displacement that comes with the exercise. In view of physical assets, respondents in all three districts reported that losing assets, such as land, houses, and shops means little hope for survival. They particularly pointed out the difficulty they go through in accessing basic services and facilities. Those who have managed to find themselves in the resettled communities bemoaned the distance to the city center and the lack of basic facilities, such as portable water supply, shopping centers, and clinics.

**Table 1:**
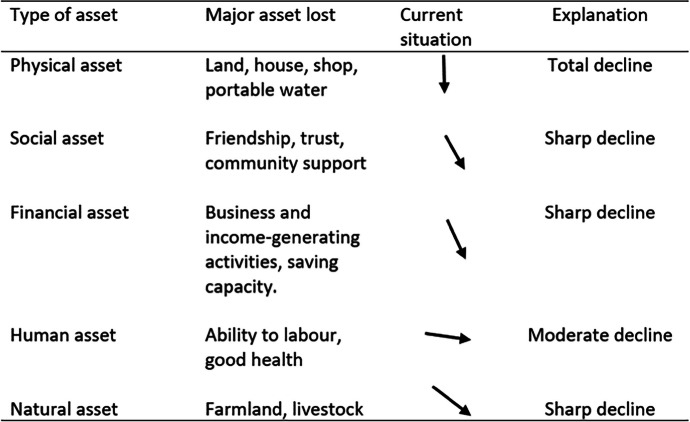
Lost assets because of expropriation in Kigali

The arrows indicate the current asset direction (total decline, sharp decline, and moderate decline). Column four explains the direction. Source: Field survey, June 2022

It emerged that some households resettled themselves at 25, 35 and 50 Kilometres away from Kigali. From the interview, it was clear that the limited access to basic infrastructure and services has triggered life dissatisfaction. A respondent from Gasabo remarked:

“The resettlement in the urban fringe results in the loss of access to some services. Everyone has access to electricity in this neighbourhood. However, access to water is very limited because the water network does not cover the whole area. Few families have connections to water pipes. Others do not have. Yet, the water supply is not regular. In addition, the access road network is not good. It is not easy to use the roads during the rain period”.

**Fig. 2:**
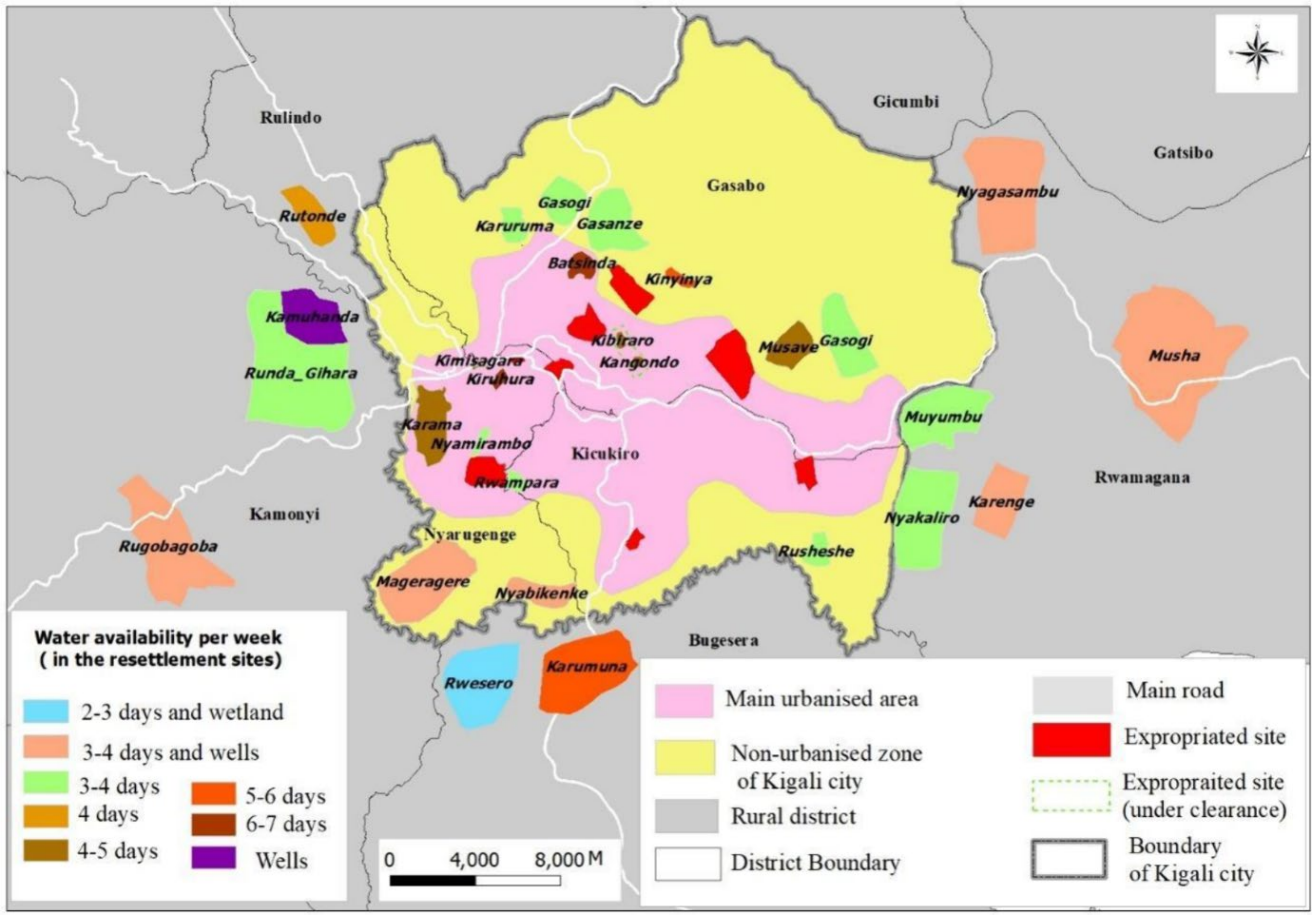
Access to water in resettled areas in Kigali. Source: Authors creation using 2022 field survey data

In the case of access to water, the study delved deeper to understand the extent to which accessibility impacts households. Figure 2 shows the status of access to water for the displaced households. These households are resettled in peripheral neighbourhoods where the supply of water is irregular (2 to 3 or 3 to 4 days per week). Irregular access to water pushes many households to resort to water from the wetlands, especially during the long dry season. Others reported using wells, which get flooded during the rainy season and dry up during the extended dry season. Many of the respondents reported deteriorating health conditions because of a lack of access to potable drinking water. This, it was made known, has added a new burden to their households.

Respondents from all the districts highlighted the hardship they are enduring all in the name of public interest. The inability of city authorities to adequately compensate them for losing their houses and lands is something they find difficult to come to terms with. A respondent Nyarugenge remarked:

“The low compensation is a threat to our ordinary urban lifestyle. We were forced to leave the city because we could not acquire new houses in the areas close to where we were living before the expropriation. In the urban periphery, we have to adapt to a new environment and lifestyle and it’s always difficult”.

The expropriation exercise consists of the compulsory taking of every private asset, including land, houses and other developments made on the land. Although this process is lawful and internationally accepted, it generally results in the loss of critical assets for the affected people. This means that due diligence is needed to minimise its impact. Losing assets means deprivation of livelihood, which many households alluded to. For households that receive some level of compensation, this means a decline in livelihood activities. The compensation which is meant for acquiring new property was reported to be always below the market value of the expropriated property and this makes it extremely difficult to acquire a new one and readjust to life. A Gasabo respondent remarked:

“Since the compensation I received was not sufficient, I could not afford a similar house in any other urban neighborhood. I was obliged to migrate towards the peri-urban area, where the house prices are lower than those of similar quality in the city”.

Another respondent from Kicukiro who is among the middle-income households stated that:

“the compensation that I received was not sufficient for acquiring another plot of land and building a new house. I was obliged to take a bank loan in order to secure the required amount for constructing another house”.

Although households can rent a small residential unit with the money, there was a general feeling of deprivation which is linked to the loss of housing ownership. People highlighted the inability to dwell in their own houses, where they could enjoy privacy and perceive security driven by the non-existence of financial burden induced by house rent. Interestingly, the loss of houses also occurs through the deprivation of other constructions made on the land. Many of the respondents, especially from Gasabo, bemoaned losing other properties (e.g., store, garden) in addition to their houses and land.

The interviews also revealed a detrimental impact on social networks. Respondents from all the districts mentioned heightened social exclusion driven by disconnection from their living and working places. This implies the loss of social networks. There was a unanimous agreement that no expropriated household disregards the friendship that they have developed over the years through settling in the same and/or close neighbourhoods or working in the same or close places. Social networks and relations are destroyed through the expropriation exercise. It emerged that every expropriated household makes its own decision about the new place to settle. This means that residents who were living in the same neighbourhood before the expropriation move to different locations and consequently break their social ties which took them so long to develop. This contributes to life dissatisfaction and an increased feeling of socio-spatial disintegration in the urban fabric. The feeling was explained by one of the respondents from Gasabo who argued as follows:

“Even though expropriated property owners use the compensation they receive in acquiring new houses in the peri-urban areas, where the costs of living may not be less hard than in Kigali, the self-relocation of displaced property owners in the remote urban outskirts is a critical challenge to sustainable well-being due to the deprivation of access to benefits like support from friends that accrue from the urban development”.

This echoes the need to revise the current compensation packages to minimize the impacts on victims. It also speaks to the need to resettle victims in the same resettled neighbourhood to minimize disruption to social networks.

The quality of neighbourhoods and availability of urban services and/or facilities contributes to people’s capacity to engage in different income-generating activities thereby enhancing the saving capacities of households. According to the interviews, all these opportunities have been lost to expropriation. Unlike in Kigali where there are numerous opportunities to tap into, the resettled communities are on the periphery and lack opportunities and support services. Respondents particularly highlighted the challenge of commuting every day to look for non-existing jobs in areas closer to Kigali. Although mention was made to self-employment initiatives, including new business ventures, and urban agriculture, among others, the needed support has not been forthcoming and this it was gathered, has made expansion and viability a big challenge. This was interesting, in the sense that people are trying to be creative amid hardship. It underscores the need for external support and the creation of entrepreneurial and vocational centres to train more people to be self-employed. Such support would go a long way to empower people, thereby reducing their dependency on the government to give them jobs.

### Asset Reconstruction Challenges

Asset reconstruction remains a big challenge to expropriated households in Kigali. One major challenge that most of the respondents alluded to is the lack of start-up capital. Many households reported their desire to invest in business activities in the new settlement areas. However, this remains a challenge as they lack the necessary initial capital for investment. Based on the interactions around compensation, it was gathered that it takes at least four months for compensation to be paid after an official notification about the expropriation activity and valuation of the concerned property. This, however, was observed to be insufficient for affected households to plan for possible investments and how to fund such. This was crucial in the sense that most of the affected people lacked readily available resources to start a new life. The situation is particularly acute for people who illegally developed their houses by squatting in unauthorised areas like wetlands under restoration. In most cases, the squatters (the majority), do not receive any compensation. Some households, especially from Gasabo, reported the challenges related to hard living conditions in their new place as follows:

“I am renting a house while before I owned my house. I currently stay home, due to the lack of job opportunities. We were paid only 40K on the first round and 20K on the second round of support for renting a house for the first two months. That is 60K in total. There has been no compensation for our property because they said that we built our houses without a permit. Developing a new business from the 60k is impossible because we have to rent and buy foodstuff. It is difficult for my family.”

Another respondent reported as follows:

“In Kigali city, I was running a small spare parts shop. I sold the items as the expropriation process was going on. I added the money to the compensation I received and built a new house. However, I was not able to save enough that would allow for running a new business. Finding opportunities for income generation is not easy after the expropriation.”

Even for those who received compensation for their houses, it was made known that the money was insufficient for investing in both new house construction and setting up a new business. One respondent from Kicukiro relies on subsistence urban agriculture in the periphery to survive. They argued that:

“Getting money is now a serious issue. It is not easy when you practice subsistence agriculture as a livelihood option”.

There was a clear sense of livelihood diversification, albeit for short-term survival and not for long-term accumulation. This is because viable livelihood options are lacking. Asked why people do not target business ventures with their compensation, it was observed that more than 60% of the expropriated households do not receive compensation that would enable them to acquire new houses and invest in viable income-generating activities. The low level of development in the urban fringes, especially the remote areas which do not offer any good employment opportunities, was highlighted by many respondents. It was observed that moving from Kigali to the periphery has not only deprived them of their life savings assets but has also put them in a state of penury and desperation, as they struggle to make ends meet. Nyarugenge respondents remarked:

“I rent a small piece of land where my wife and I practise subsistence agriculture two times a year. I am a land market solicitor. I also do construction work. However, the land market is not functioning well due to the very limited number of buyers. The construction sector is also not developed in the area”.

This argument was supported by other respondents as follows:

“I try to find some employment opportunities like in the construction sector. However, they are very limited here”.

“I have no job in general. I am at home. My husband does some casual jobs like an assistant mason. He is also sometimes hired for the cultivation of some farmers’ land. What we earn is not sufficient so we do not have any other resource that we may invest in the activities that generate the income”.

“My wife and I had a small food shop from which we could earn around 150K (Rwandan Franc) per month before the expiration. Today, we are running the same business. But there are not sufficient customers in this area since it is a rural area where some people produce some of their food products. Our income has decreased since we cannot earn more than 100K per month”.

This echoes that expropriated households are engaged in precarious jobs. Thus, the lack of sufficient funds for the resettlement processes and investment options for improving livelihood makes the living conditions of expropriated households difficult. This puts them in a vulnerable situation and calls for the need to think more deeply about future expropriation activities. Alternative livelihood options must be part and parcel of future initiatives to restore hope and make people more optimistic about life after expropriation.

**Fig. 3:**
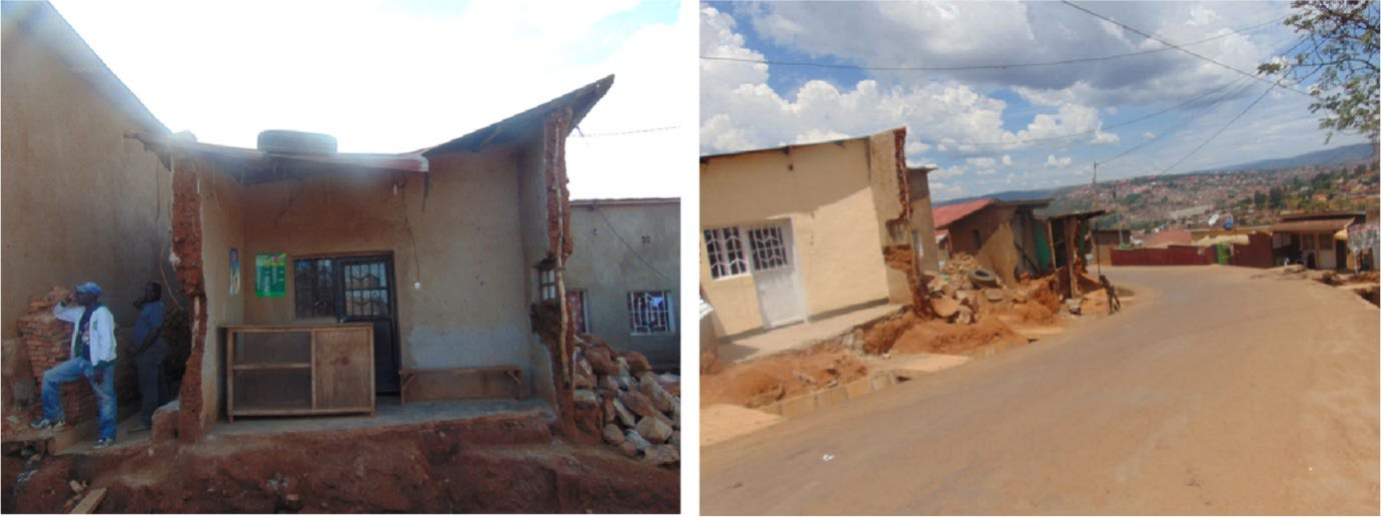
Partially expropriated houses in Kigali. Source: Field survey, July 2019

Many respondents also mentioned the incapacity to rehabilitate partially expropriated properties as a major post-expropriation asset reconstruction challenge. This stems from the fact that in most cases they get little compensation for their property. Almost 40 percent of the respondents reported this as being worrying but with no option to change the practice. They argued that the partial destruction of a property like a house, results in little payment of money which is always insufficient for the reconstruction of the destroyed part of the house. Figure 3 show partial expropriation in one of the affected areas. Commenting on this case, a Gasabo respondent remarked:

“During the construction of the road, they compensated the part of my house that was affected by the road. The compensation was too little, as it was only 3 million Rwandan Francs. My family was required to take a loan to rehabilitate the remaining part of the house. In terms of expropriation, there has been a loss. However, considering the benefit of being on the main road, the house has more value. The expropriated family suggests that the compensation should be increased in such circumstances. My wish is that they could expropriate the whole property”.

Though the respondent was happy as the value of his house increased due to its proximity to the road, it is not always the case for most of the people whose properties are affected. However, this was an interesting finding in the sense that property value was also dependent on proximity to major public construction. It emerged that in most cases, properties that are closer to main public facilities tend to be valued more than those further away and, hence, receive higher compensation.

### Livelihood Coping Strategies

Given the challenges that people are going through to reconstitute their assets, the study further investigated livelihood coping strategies. Three major activities emerged from the interviews: urban agriculture, small-scale non-farm businesses and migration. In a bid to survive in their new environment, many households have resorted to growing crops and vegetables and rearing livestock (e.g., pigs, goats, and poultry) to make a living. Although this was a departure from their original livelihood activities in Kigali, the new environment was a perfect fit for such activities. However, the scarcity of land in the periphery means that these activities are conducted on a small plot of rented land. The desire to increase production has pushed many people to negotiate land leases from original inhabitants who own large plots of uncultivated lands. This has been the situation with at least fifty percent of the respondents from all the districts.

Interestingly, those who have not been able to cope with the conditions in the new sites have been migrating to other secondary cities and nearby towns and villages for livelihood opportunities. Other activities that have become popular among residents include operating (mini) provisions stores and drinking sports, sewing, and masonry work. One observation was that people are combining different activities at the same time. This is livelihood diversification, and it is important for smoothing consumption and spreading risk. Livelihood diversification is what keeps people alive and is the main resilience building block in the new environment. A respondent from Gasabo remarked:

“You can’t live here doing just one thing; no, it is not possible. You have to work hard and get money from different sources to support your family. That is what I do, my friends are also doing it; in fact, everyone here”.

This respondent has a small garden in addition to working in the industrial zone around the inner city. Another respondent from Nyarugenge alluded to what his family is doing to survive:

“My wife is engaged in the sale of local food products. I provide washing services to different people in my former neighbourhood and leasing two small houses”.

### Expropriation Opportunities

Although largely perceived to be negative in terms of socioeconomic impacts, a fairly compensated expropriation process comes with lots of positives, including a change in the quality of life and new business opportunities. From the interviews, it emerged that those who received adequate compensation for their properties are living a better life since they were able to acquire new properties in the city periphery or move to a better neighbourhood in the city. Those who moved to the fringes were forced because of the price of plots and housing construction in the city.

**Fig. 4:**
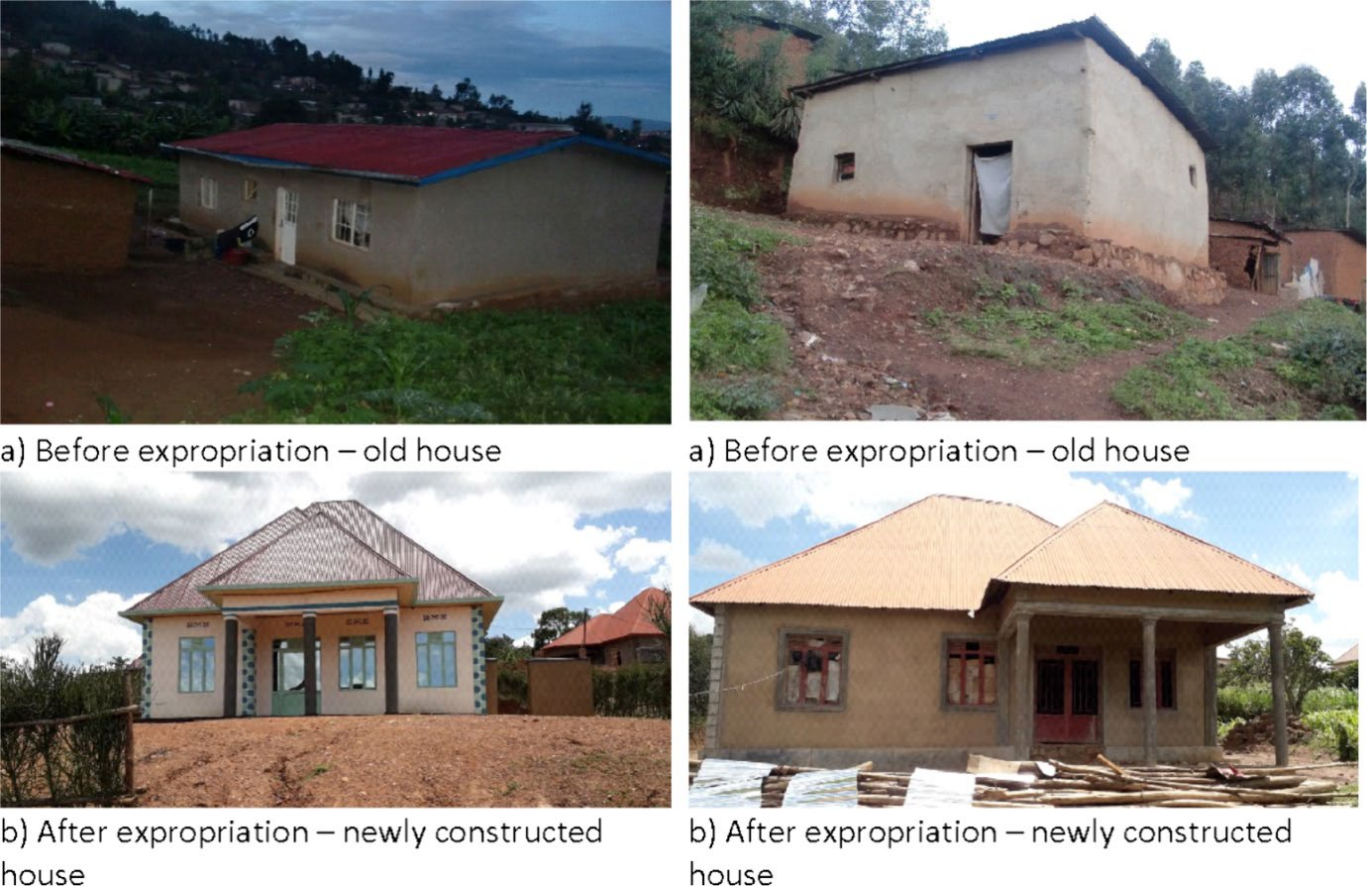
Old and new houses of expropriated households. Source: Field survey, July 2019

It was revealed that although building a new house in the periphery is less expensive (between 8,000 and 15,000 USD) compared to Kigali city (above 25,000 USD), most of the newly built houses are of better quality than those which were expropriated as shown in Fig. 4 below. Figure 4 shows two housing situations (before and after) of two households that were expropriated in 2019. The newly constructed houses are of better quality in all areas, including size, construction material and usability. They also fit into the housing code of the zoning area which sits under the Kigali City Master Plan. Interestingly, there was unanimous agreement among the respondents that receiving fair compensation is key to a better life in or outside the city. This is a clear opportunity for social mobility and underscores the need to review the current compensation package to be more comprehensive.

Another major opportunity is using part of the compensation for lucrative businesses. Again, this is also the case for those who received sufficient compensation for their properties. Unfortunately, most of the people interviewed do not fall into this category, suggesting that most of the expropriated households are poor households who were illegally occupying strategic areas in Kigali city. This partly explains why they receive little compensation. Notwithstanding, legal occupants expropriated from Kigali have seen significant improvement in their living conditions through booming small-scale businesses and living in modern houses. One respondent from Gasabo highlighted how his life has changed because of the new opportunities in the new environment.

“My life is better now, I can’t complain. I have a new house and I’m also operating a business which is running well.”

This household received sufficient compensation for two properties: one for a dwelling and the other for rented property. Using the compensation for one of the houses, they bought land and constructed a dwelling unit in the newly resettled neighbourhood. The other compensation was used to establish a modest spot. The respondent mentioned that this investment has been a life changer in that it fetches a lot of money for their household and life is now better compared to when they were in Kigali city. However, there was an admission that things were not easy in the first year due to the limited number of customers, low population density in the area, and the limited social integration as they were perceived as strangers. However, things started picking up as more people moved to the area and people began to develop social relationships in the area. Other respondents also reported doing well in other newly found businesses such as retail and sewing. Regardless, one thing that stood out from the interviews was the need for financial support to expand ongoing businesses. Evidently, the provision of viable business options and appropriate vocational training in these resettled neighbourhoods by the government could help improve the livelihood of expropriated households.

## Discussion

### Asset Deprivation, Unfair Compensation and Poverty

The study's central focus revolves around the nuanced impact of land expropriation on asset endowment. The findings reveal a concerning trend of asset deprivation, primarily affecting vulnerable populations. The loss of critical assets such as land, houses, and stores, exacerbated by compensation which does not allows for the livelihood reconstitution, pushes affected individuals into a precarious situation, with risks for deepening the cycle of poverty. This echo existing literature emphasising the pivotal role of assets in providing economic stability and reducing vulnerability (Baffoe & Matsuda, 2017; Nel, 2015). Similarly, Hudani (2020) argues that the expropriation results in the crisis constituting a visceral rift in the everyday and a rupture of the everyday existence of the affected people when they struggle for remaining within the bounds of the city. A lack of assets undermines successful engagement, a situation that exposes one to all manner of shocks. Moser (1998, p 3) argued that ‘‘the more assets people have, the less vulnerable they are, and the greater the erosion of people’s assets, the greater their insecurity.’’ A lack of productive assets deprives people of their livelihood, thereby plunging them into extreme poverty, and this is the case in Kigali. Residents in the newly resettled neighbourhoods outside of Kigali increasingly suffer in an effort to reconstitute their assets to enable them to engage in meaningful livelihood. This is partly explained by the lack of employment opportunities and unjust compensation for expropriated properties. The present situation of destitution and hopelessness lend support to existing literature that access to assets, especially physical assets, particularly in the urban context, affords the poor the opportunity to be economically viable, thereby escaping poverty (Fontana, 2016). Assets are critical for poverty reduction, particularly in the urban informal context, because most residents of informal settlements are unsalaried workers, and the ability to improve their well-being is contingent upon their access and accumulation of diverse assets and the return they generate (Moser & Dani, 2008).

While access to essential and basic urban facilities offers different benefits, including enjoyment of urban life, the increased distance to basic urban facilities and services presents expropriation (driven by the rapid urban transformation of Kigali) as a threat to livelihood. As pointed out by Goodfellow (2014, 2022) the loss of access to basic urban infrastructure and services through displacement increasingly contributes to life dissatisfaction and a rising feeling of spatial disintegration which are commonly expressed by residents in Kigali these days. The lack of critical assets also suggests deprivation of opportunity to engage in the labour market. The loss of income generating opportunities has been a major problem among affected households in Kigali and a key factor underlying deteriorated quality of life in the city. Loss of social assets, for instance, is blamed for the increasing level of social disintegration which is gradually impacting the mental health of residents. The destruction of social ties and resettlement in different locations as a result of expropriation is consistent with previous studies in the same context (Nikuze, et al., 2019; Uwayezu & de Vries, 2019). Where an expropriated household finally settles is largely determined by the availability of alternative livelihood options, price of land and distance to the city centre. Interestingly, the preference has been to live closer to the city centre and this is explained by the proximity to schools, bus service, shops and better job prospects. However, in practice, it is fuelling the emergence of more informal settlements and uncontrolled growth. This is because most of the expropriated households are informal dwellers who receive little compensation which is always insufficient to acquire new property in a formal neighbourhood or even in the periphery (Nduwayezu et al., 2021). Their best bet, therefore, is to move to a temporary space within the city, with another expropriation staring at them. The current practice defeats the whole aim of the expropriation exercise and begs the question if due diligence was carried out before embarking on such activities. The practice of unjust compensation is particularly problematic and explains why most affected people are struggling to survive. Although the international recommendation is that compensation must be just to allow people to rebuild their livelihood (Gutwald et al., 2014), this is not always followed in the scheme of things in Kigali. The current practice leaves much to be desired and it is a wake-up call for the city authorities, especially if the attainment of SDG 11 – Make cities inclusive, safe, resilient, and sustainable – is anything to go by. The current outlook dents the image of the city as a “Model City of Africa”. It is suggested by the expropriation expert groups that for expropriation to be fair, it must always be preceded by a transparent and participatory procedure (Tagliarino et al., 2018). This is important because a well-exercised power of eminent domain has every potential to promote economic development and job creation (World Bank, 2015a, b).

The lack of investment in critical urban infrastructure coupled with non-participatory planning approaches can undermine the development of inclusive governance and there is a risk that this can happen in Kigali. The asset dispossession and attendant challenges findings support high-level reports about expropriation and urban development in African cities (UN-Habitat, 2014, 2016; World Bank, 2018). Clearly, an integrated approach involving investment in critical urban infrastructure and services, inclusive planning, just compensation and alternative livelihood options, and training programmes are needed to address asset deprivation in Kigali.

### Defying the Odds: Surviving Amidst Hardship

Expropriation poses serious challenges, including livelihood disruption and loss of productive assets. These challenges put the expropriated households in a precarious situation and for those who receive little compensation, it is a matter of surviving at all costs. In the new resettlement areas, people adopt different options in a bid to reconstitute their livelihoods. They follow the trajectory of livelihood diversification, which is known to be important in building shock absorbers, especially among poor people (Ellis, 2000; Moreda, 2023; Baffoe & Matsuda, 2018). Although most of these options, including urban agriculture, and casual work are highly vulnerable, one cannot disregard their relevance in spreading risk and smoothing consumption, and building local resilience in the resettled neighbourhoods. For many, combining different livelihood options is unnegotiable as it is the main strategy to cope with the increasing economic hardship. This speaks to the thesis of livelihood priority and viability in vulnerable environments. Baffoe and Matsuda (2017) argued that priority is not equivalent to viability. They explained that while the former speaks to the necessity to engage in a particular livelihood activity for survival purposes, the latter critically considers the economic value and accumulation potential of the activity. Although people are coping, more viable livelihood options are needed for adaptation, asset accumulation, and resilience building in the newly resettled communities. In these areas, those who were previously relying on subsistence agriculture and casual work in the previous neighbourhoods have diversified to growing food crops and livestock breeding in the periphery. However, there is a scarcity of farmland in the peri-urban areas due to rapid transformation. This means that the farming activity is carried out on a small piece of land, and this is explained by a high level of fragmentation driven largely by traditional practices of ascending partitioning that has been one of the modes of access to land in Rwanda (Ntihinyurwa et al., 2019). It is also underscored by the limited purchasing power of most expropriated households as many use parts of the compensation to acquire new houses and for subsistence. Overall, however, people are using diverse means, albeit less profitable, to counter the hardship engineered by expropriation.

### The Potential of Expropriation in Improving Wellbeing

Regardless of the socio-economic ramifications, a well-executed expropriation exercise comes with lots of benefits, including boosting economic growth (World Bank, 2018), environmental conservation, development of biological species, (Chen et al., 2019; Debonne et al., 2019), improved housing quality and quality of life. In Kigali, this study has revealed that expropriation does not always result in material deprivation or livelihood hardship. When due process is followed and just compensation is paid, expropriation offers the opportunity to move to better housing and invest in viable business ventures. Adequate compensation means that affected people can use the money to better lives, which has been the case for some of the respondents in the present case. This study has demonstrated that just compensation has the potential to drive social mobility. Access to better housing and new business opportunities are key drivers that can push lower-income households up the social ladder by improving their living conditions. The findings indicate that those who received sufficient compensation are better off compared to their counterparts with compensation which does not allow for accessing other properties. The excitement that people expressed for their new life is evidence that a well-executed expropriation could be a game changer for the vulnerable group. In Kigali, the cost of land and housing development decreases as the distance from the city center to the urban fringe increases. The decrease in prices constitutes an opportunity for those with just compensation to develop a new property, putting them in a genuine position to transform their life. To make this transformation sustainable, however, it is important to invest more in affordable housing and basic infrastructure and implement a participatory resettlement process. Also worthy of attention is the creation of employment opportunities, especially investment in small-scale businesses and technical and vocational educational training (TVET) programmes and the provision of quality education.

## Conclusion

In this study, we seek to understand how the implementation of land expropriation in Kigali City has impacted asset endowment and well-being. Our analysis has shown that expropriation deprives the expropriated property owners of productive assets, including land, houses and stores. The loss coupled with unjust compensation, we argue, tends to expose the affected people who are largely vulnerable to a trap of poverty, making life unbearable for them. The practice, the study further argues, dents the image of Kigali as a “Model City of Africa”. In adjusting to life in the resettled neighbourhoods, livelihood diversification has become the main resilience-building strategy. Although most of the activities appear not too economically viable, they form the pillar of new livelihood as they play an important role in spreading risk and smoothing consumption, particularly among poor households. For most expropriated households, concurrently engaging in different income-generating activities regardless of their complexities is the only adaptation strategy readily accessible to them. Interestingly, the study further revealed that expropriation does not always result in material deprivation or livelihood hardship. In cases where just compensation is paid, expropriation appears to be a driver of social mobility through access to better housing and investment in profitable businesses. Thus, adequate compensation offers affected households the opportunity to better their lives, which has been the case for some of the respondents in this study. The study has provided evidence to demonstrate that adequate compensation has the potential to drive social mobility. Access to better housing and new business opportunities are key drivers that can propel lower-income households up the social ladder by improving their living conditions. The findings indicate that those who received sufficient compensation for their properties are living an improved life compared to their counterparts with compensation which does not allow for accessing other properties. From the analysis, it is evident that expropriation has significant socio-economic impacts, and addressing these would require integrated and multifaceted measures. Addressing asset deprivation would require revision of the current compensation package by the government to be more just to enable affected households to start a [decent] new life. It is also important for the government, particularly Kigali City Administration, to invest in affordable housing and basic infrastructure, but also a well-designed resettlement programme implemented in collaboration with affected property owners and other relevant stakeholders, such as civil society groups and international development partners. In a similar vein, the government should consider the provisioning of alternative viable livelihood options and the promotion of diversification by accumulation. More so, investment in small-scale businesses and establishing technical and vocational educational training (TVET) centers to train local entrepreneurs would be key. Effectively championing these measures by the government would go a long way to leverage the benefit of expropriation, including reinforcing the social mobility potential. Kigali has the potential to redefine its approach to expropriation and realize the vision of being a truly inclusive and sustainable Model City of Africa. The findings presented here underscore the importance of continued empirical analysis to refine and reinforce our understanding of these complex dynamics.

## References

[CR1] Bafana, B. (2016). Kigali sparkles on the hills. Africa Renewal. https://www.un.org/africarenewal/magazine/april-2016/kigali-sparkles-hills. Accessed 3 Apr 2023.

[CR2] Baffoe, G. (2023). Neoliberal urban development and the polarization of urban governance. *Cities,**143*, 104570. 10.1016/j.cities.2023.104570

[CR3] Baffoe, G., & Matsuda, H. (2017). Why do rural people do what they do in the context of livelihood activities? Exploring the Livelihood Viability and Priority Nexus. *Community Development,**48*(5), 715–734. 10.1080/15575330.2017.1366927

[CR4] Baffoe, G., & Matsuda, H. (2018). An empirical assessment of rural livelihood assets from gender perspective: Evidence from Ghana. *Sustainability Science,**13*, 815–828. 10.1007/s11625-017-0483-8

[CR5] Baffoe, G., Malonza, J., Manirakiza, V., & Mugabe, L. (2020). Understanding the concept of neighbourhood in Kigali City. *Rwanda. Sustainability,**12*, 1555. 10.3390/su12041555

[CR6] Baffoe, G., & Roy, S. (2022). Colonial legacies and contemporary urban planning practices in Dhaka, Bangladesh. *Planning Perspective*, 1–24. 10.1080/02665433.2022.2041468

[CR7] Bao, H. J., Fang, Y., & Lei, P. (2016). Conflict of interest in land acquisition: Behavioral selection mechanism and empirical evidence of local governments and land-losing farmers. *China Land Science,**30*(37), 21–27.

[CR8] Bernahu, G., & Woldemikael, S. M. (2022). The interrelationships of sustainable livelihood capital assets deprivations and asset based social policy interventions: The case of Addis Ababa informal settlement areas. *Ethiopia. Research in Globalization,**4*, 100081. 10.1016/j.resglo.2022.100081

[CR9] Boateng, K. P. (2013). A livelihood assets status tracking method for the assessment of the effects of a development programme on agricultural productivity and poverty reduction: evidence from the Ejisu-Juabeng District, Ghana. *Journal of Agriculture and Environment for International Development* 107(2):267–296. 10.12895/jaeid.20132.181

[CR10] Brunting, E., Steele, J., Keys, E., Muyengwa, S., Child, B., & Southworth, J. (2013). Local perception of risk to livelihoods in the semi-arid landscape of Southern Africa. *Land,**2*, 225–251. 10.3390/land2020225

[CR11] Castro-Arce, K., & Vanclay, F. (2020). Community-led green land acquisition: social innovative initiatives for forest protection and regional development. *Land,**9*(4), 109. 10.3390/land9040109

[CR12] Chen, B., Kennedy, C. M., & Xu, B. (2019). Effective moratoria on land acquisitions reduce tropical deforestation: Evidence from Indonesia. *Environmental Research Letters,**14*, 044009. 10.1088/1748-9326/ab051e

[CR13] Creswell, J. W. (2009). *Research Design, Qualitative, Quantitative, and Mixed Methods Approaches* (3rd ed.). Sage Publications Inc.

[CR14] Daily, G., Kareiva, P., Polasky, S., Ricketts, T., & Tallis, H. (2011). Mainstreaming natural capital into decisions. In H. Tallis, T. Ricketts, G. Daily, S. Polasky, & P. Kareiva (Eds.), *Natural capital: Theory and practice of mapping ecosystem services* (pp. 3–14). Oxford University Press.

[CR15] de Bruin, S., Dengerink, J., & van Vliet, J. (2021). Urbanisation as driver of food system transformation and opportunities for rural livelihoods. *Food Security,**13*, 781–798. 10.1007/s12571-021-01182-8 34221191 10.1007/s12571-021-01182-8 PMC8237550

[CR16] De Schutter, O. (2010). The emerging human right to land. *International Community Law Review,**12*(3), 303–334. 10.1163/187197310X513725

[CR17] Debonne, N., van Vliet, J., & Verburg, P. (2019). Future governance options for large-scale land acquisition in Cambodia: Impacts on tree cover and tiger landscapes. *Environmental Science and Policy,**94*, 9–19. 10.1016/j.envsci.2018.12.031

[CR18] Ding, C. (2007). Policy and praxis of land acquisition in China. *Land Use Policy,**24*, 1–13. 10.1016/j.landusepol.2005.09.002

[CR19] Ding, W., Jimoh, S. O., Hou, Y., Hou, X., & Zhang, W. (2018). Influence of Livelihood Capitals on Livelihood Strategies of Herdsmen in Inner Mongolia. *China. Sustainability,**10*, 3325. 10.3390/su10093325

[CR20] Ellis, F. (2000). *Rural livelihoods and diversity in developing countries*. Oxford University Press.

[CR21] Esiara, K. (2015). Shortage of low-cost housing to continue biting in Kigali. A featured article on The EastAfrican. https://www.theeastafrican.co.ke/rwanda/Business/Shortage-of-low-cost-housing-to-continue-biting-in-Kigali/1433224-2607494-o9vxhjz/index.html (Accessed 3 December 2022).

[CR22] Focus on land in Africa 2019. Land Rights and Development in Rwanda. http://www.focusonland.com/countries/rwanda/ (Accessed 12 March 2023).

[CR23] Fontana, C. (2016). Hernando de Soto on Land Titling: Consensus and Criticism. *Next Generation Planning,**3*, 36–48. 10.24306/plnxt.2016.03.003

[CR24] Goodfellow, T. (2014). Rwanda’s political settlement and the urban transition: Expropriation, construction and taxation in Kigali. *Journal of Eastern African Studies,**8*(2), 311–329. 10.1080/17531055.2014.891714

[CR25] Goodfellow, T., & Smith, A. (2013). from urban catastrophe to “Model” City? Politics, security and development in post-conflict Kigali. *Urban Studies,**50*(15), 3185–3202. 10.1177/0042098013487776

[CR26] Goodfellow,T. (2022). Politics and the Urban Frontier: Transformation and Divergence in Late Urbanizing East Africa. London: Oxford University Press; p. 336.

[CR27] Government of Rwanda. Law N° 32/2015 of 11/06/2015 Relating to Expropriation in the Public Interest; Government of Rwanda: Kigali, Rwanda, p. 54.

[CR28] Gummesson, E. (1988). *Qualitative methods in management research*. Lund, Norway: Studentlitteratur, Chartwell-Bratt.

[CR29] Gutwald, R., Leßmann, O., Masson, T., & Rauschmayer, F. A. (2014). Capability Approach to Intergenerational Justice? Examining the Potential of Amartya Sen’s Ethics with Regard to Intergenerational Issues. *Journal of Human Development and Capabilities,**15*, 355–368. 10.1080/19452829.2014.899563

[CR30] Hudani, S. E. (2020). The green masterplan: crisis, state transition and urban transformation in post-genocide rwanda. *International Journal of Urban and Regional Research,**44*, 673–690. 10.1111/1468-2427.12910

[CR31] Landesa Rural Development Institute. (2012). Findings from Landesa’s survey of rural China published. https://www.landesa.org/press-and-media/6th-china-survey/. Accessed 21 Jan 2023.

[CR32] Landesa Rural Development Institute. (2018). 17-province survey on farmers’ land rights in China, 2016. https://www.landesa.org/resources/17-province-survey-on-farmers-land-rights-in-china-2016/. Accessed 21 Jan 2023.

[CR33] Li, Z. L., & Hu, Z. H. (2018). On the contractual solution of the dispute over the compensation standard for the expropriation of agricultural land. *Journal of China University of Geosciences (Soc. Sci. Ed.), 18*, 168–177.

[CR34] Li, X., Lu, S. G., & Wang, H. (2019). Research on the impact of land acquisition on farmers’ income and its spatial differentiation—based on the analysis of the difference method of CHFS Data. *China Land Science,**33*, 102–110.

[CR35] Lu, Y., & Xu, Y. (2016). A geographic identification of multidimensional poverty in rural China under the framework of sustainable livelihoods analysis. *Applied Geography,**76*, 62–76. 10.1016/j.apgeog.2016.06.004

[CR36] Ma, H. S. Zhao, O. & Gao, J. X. (2016). Discussion on land acquisition compensation distribution based on agricultural land development rights. *Journal of Engineering Management, 30*, 44–48.

[CR37] Manirakiza V., Mugabe L., Nsabimana A. & Nzayirambaho M. (2019). City Profile: Kigali, Rwanda. *Environment and Urbanisation ASIA, 10*(2), 290–307. https://journals.sagepub.com/doi/abs/10.1177/0975425319867485

[CR38] Marco-Thyse, S. (2006). Land rights in South Africa: A mechanism against poverty? *Development,**49*, 133–137. 10.1057/palgrave.development.1100290

[CR39] Moreda, T. (2023). The social dynamics of access to land, livelihoods and the rural youth in an era of rapid rural change: Evidence from Ethiopia. *Land Use Policy,**128*, 106616. 10.1016/j.landusepol.2023.106616

[CR40] Morse, S., & McNamara, N. (2013). *Sustainable livelihood approach: A critique of theory and practice*. Springer Science and Business Media.

[CR41] Moser, C. O. N. (1998). The asset vulnerability framework: Reassessing urban poverty reduction strategies. *World Development, 26*(1), 1–19. 10.1016/S0305-750X(97)10015-8

[CR42] Moser, C., & Dani, A. A. (2008). *Assets, Livelihoods and Social Policy*. World Bank Group. 10.1596/978-0-8213-6995-1

[CR43] National Institute of Statistics of Rwanda, (2023). Fifth Rwanda Population and Housing Census (RPHC5). Main Indicators Report; Kigali, Rwanda. p. 178.

[CR44] Nduwayezu, G., Manirakiza V., Mugabe, L. & Malonza, J. (2021). Urban Growth and Land Use/Land Cover Changes in the Post-Genocide Period in Kigali, Rwanda. *Environment and Urbanisation ASIA*, 12(1 Supplementary). 10.1177/0975425321997971

[CR45] Nel, H. (2015). An integration of the livelihoods and asset-based community development approaches: A South African case study. *Development Southern Africa,**32*(4), 511–525. 10.1080/0376835X.2015.1039706

[CR46] Nikuze, A., Sliuzas, R., Flacke, J., & van Maarseveen, M. (2019). Livelihood impacts of displacement and resettlement on informal households - a case study from Kigali, Rwanda. *Habitat International,**86*, 38–47. 10.1016/j.habitatint.2019.02.006

[CR47] Ntihinyurwa, P. D., de Vries, W. T., Chigbu, U. E., & Dukwiyimpuhwe, P. A. (2019). The positive impacts of farmland fragmentation in Rwanda. *Land Use Policy,**81*, 565–581. 10.1016/j.landusepol.2018.11.005

[CR48] OECD. (2013). How’s life? 2013: measuring well-being. OECD Publishing.10.1787/23089679

[CR49] Ong, L. H. (2020). Land grabbing in an autocracy and a multi-party democracy: China and India compared. *Journal of Contemporary Asia,**50*, 361–379. 10.1080/00472336.2019.1569253

[CR50] Pu, S. G., & Chen, G. (2009). Thoughts on solving the problem of urban land acquisition and demolition. *Economic Research Ref.,**71*, 60–66.

[CR51] Republic of Rwanda. (2015). Law N.32/2015 of 11/06/2015 relating to expropriation in the public interest. Official Gazette N.35 of 31/08/2015. Kigali: Government of Rwanda.

[CR52] Reyntjens, F. (1980): C. L. R, Vol. II, Butare, UNR, p. 310.

[CR53] Rose, H., Mugisha, F., Kananga, A. & Clay, D. (2016). The Implementation of Rwanda’s Expropriation Law and Its Outcomes on The Population. In Proceedings of the 2016 World Bank Conference on Land and Poverty, Washington, DC, USA. https://www.land-links.org/research-publication/the-implementation-of-rwandasexpropriation-law-and-its-outcomes-on-the-population/ (Accessed on 19 February 2023).

[CR54] Rwanda Civil Society Platform. (2017). Analysis of land expropriation and transfer process in Rwanda. Final Report. Kigali, Rwanda. http://www.rcsprwanda.org/IMG/pdf/report_on_land.pdf (Accessed 21 December 2022).

[CR55] Soma, H., Sukhwani, V., & Shaw, R. (2020). An approach to determining the linkage between livelihood assets and the housing conditions in urban slums of Dhaka. *Journal of Urban Management*. 10.1016/j.jum.2021.08.006

[CR56] Tagliarino, N. K., Bununu, Y. A., Micheal, M. O., de Maria, M. & Olusanmi, A. (2018). Compensation for expropriated community farmland in Nigeria: An in-depth analysis of the laws and practices related to land expropriation for the lekki free trade zone in Lagos. *Land, 7*(1), 23. 10.3390/land7010023

[CR57] Tura, H. A. (2018). Land use policy land rights and land grabbing in Oromia, Ethiopia. *Land Use Policy,**70*, 247–255. 10.1016/j.landusepol.2017.10.024

[CR58] UN-Habitat. (2014). Urbanization and Development: Emerging Futures. Retrieved from https://unhabitat.org/urbanization-and-development-emerging-futures (Accessed 30 January 2023). C:/Users/User/Downloads/UNDP_RBLAC_Livelihoods%20Guidance%20Note_EN-210July2017.pdf.

[CR59] UN-Habitat. (2016). Urbanization and Development: Investing in Livelihoods, Environment and Infrastructure. Retrieved from https://unhabitat.org/urbanization-and-development-investing-in-livelihoods-environment-and-infrastructure (Accessed 30 January 2023).

[CR60] UNDP. (2017). Application of the sustainable livelihood frameworks in development projects. Panama City: United Nations Development Program (UNDP). C:/Users/User/Downloads/UNDP_RBLAC_Livelihoods%20Guidance%20Note_EN-210July2017.pdf

[CR61] USAID. (2014). LAND Project, Research Brief No.2, Kigali.

[CR62] Uwayezu, E., & de Vries, W. T. (2019). Expropriation of Real Property in Kigali City: Scoping the Patterns of Spatial Justice. *Land,**8*, 23. 10.3390/land8020023

[CR63] Uwayezu, E., & de Vries, W. T. (2020). Can In-Kind Compensation for Expropriated Real Property Promote Spatial Justice? A Case Study Analysis of Resettlement in Kigali City, Rwanda. *Sustainability,**12*, 3753. 10.3390/su12093753

[CR64] Wang, Y., Li, W., Xiong, J., Li, Y., & Wu, H. (2019). Effect of land expropriation on land-lost farmers’ health: empirical evidence from Rural China. *International Journal of Environmental Research and Public Health,**16*, 2934. 10.3390/ijerph16162934 31443280 10.3390/ijerph16162934 PMC6720733

[CR65] Wang, M., Li, M., Jin, B., Yao, L., & Ji, H. (2021). Does livelihood capital influence the livelihood strategy of Herdsmen? *Evidence from Western China. Land,**10*, 763. 10.3390/land10070763

[CR66] Wineman, A., & Jayne, T. S. (2017). Land Prices Heading Skyward? An Analysis of Farmland Values across Tanzania. *Applied Economic Perspective and Policy,**40*(2), 187–214. 10.1093/aepp/ppx038

[CR67] World Bank. (2015a). Expropriation. https://urban-regeneration.worldbank.org/node/34. Accessed 30 Jan 2023.

[CR68] World Bank. (2015b). Environmental and Social Standard 5 Land Acquisition, Restrictions on Land Use and Involuntary Resettlement. Washington, DC., USA. p. 13.

[CR69] World Bank. (2018). Poverty and Shared Prosperity 2018: Piecing Together the Poverty Puzzle. Retrieved from https://openknowledge.worldbank.org/handle/10986/30416 (Accessed 24 January 2023).

[CR70] World Bank 2020. Urban Development. https://www.worldbank.org/en/topic/urbandevelopment/overview (Accessed 24 January 2023).

[CR71] World Cities Report. (2020). The value of sustainable urbanization. UN-Habitat, Nairobi, Kenya. https://unhabitat.org/sites/default/files/2020/10/wcr_2020_report.pdf (Accessed 30 January 2023).

[CR72] Yin, R. K. (2009). *Case study research: Design and methods* (4th ed.). Library of Congress Cataloguing-in-Publication Data.

[CR73] Zhao, X., Jin, L., & Sun, S. B. (2022). Gone with the land: Effects of land expropriation on health and subjective well-being in rural China. *Health and Place,**73*, 102614. 10.1016/j.healthplace.2021.102614 34246530 10.1016/j.healthplace.2021.102614

[CR74] Zhao, X. & Xie, Y. (2022). The effect of land expropriation on local political trust in China. *Land Use Policy, 114*, 105966. 10.1016/j.landusepol.2021.105966

